# Ten Simple Rules for Providing a Scientific Web Resource

**DOI:** 10.1371/journal.pcbi.1001126

**Published:** 2011-05-26

**Authors:** Sebastian J. Schultheiss

**Affiliations:** Machine Learning in Biology Research Group, Friedrich Miescher Laboratory of the Max Planck Society, Tübingen, Germany; University of California San Diego, United States of America

Many projects in computational biology lead to the creation of a small application program or collection of scripts that can be of use to other scientists. A natural progression is to make this tool available via a Web site or by creating a service for it, from now on collectively called “Web resource.”

We conducted a survey among providers and users of scientific Web resources, as well as a study on availability. The following rules reflect the experiences and opinions of over 250 scientists who have answered our questions and who use Web resources regularly, as well as our own experience. The study of availability allows us to draw objective conclusions about the characteristics of those Web resources that are still available and correlate the features that distinguish them from disappeared or nonfunctional ones. These ten simple rules aid you in designing and maintaining a scientific Web resource that is available to anyone interested in using it.

## Rule 1: Plan Your Resource

As soon as you are seriously thinking about offering a Web resource to the general public, it is a good idea to lay down some ground rules. Clarify responsibilities in the processes of developing and maintaining the resource. Discuss these issues with the senior author or principal investigator, who is ultimately responsible for the availability of the resource. Read more about some ideas to manage responsibility in Rule 2.

Try to think of a good name that is not already taken and can be easily remembered. Changing the Web address of an existing resource is hard to do; it's better to start off with your own Internet domain name or a persistent URL. For the latter, the Online Computer Library Center offers a Persistent Uniform Resource Locator (PURL) for a changing Web address (for an overview, see [1]). It is essentially a transparent link to wherever your resource is currently hosted; its destination can be updated accordingly.

Some decisions early on can greatly impact the resource over its whole life cycle. Consider the level of service you want to offer. Is it a simple tool one step up from a command-line interface or a whole framework for large-scale analysis? How will users be able to access it? Read more about these options and how to make good use of the infrastructure available to you in Rule 4.

Throughout the life of your resource, there may be many different people involved in developing and maintaining it. Documentation is important for both developers and users of the resource. A scientific Web resource should be offered as open source software. Making your resource a software project at SourceForge.net, for instance, greatly facilitates development and maintenance. This also lets you keep an open channel of communications with your users, tell them about any major changes, and get their feedback to shape future developments.

Eventually, the resource may have outlived its usefulness. Read Rule 10 to find out when and how to shut down operations.

## Rule 2: Discuss Responsibilities

More than 58% of resources are developed entirely by researchers without a permanent position who will eventually move to another institution.

As a graduate student, involve your advisors early when you consider providing a Web resource. Chances are, they already know a way to share the work load. Discuss the issue of software maintenance, both for the time the original developers are still on site and for the time they have moved on. Do you want to take your work with you or leave it behind?

As an advisor, remember that this issue could come up, at the latest when your student leaves. As the senior author, solving such issues are your responsibility. Feel free to direct students towards using a certain software framework; creating such lab rules limits responsibility in a good way. You can even think of creating an intergenerational treaty for software maintenance among students in different years.

If your resource is used by collaborators and they think your program is valuable enough, you could convince them to take it over. The same is true for one of the following institutions: If your resource has a high impact and is useful to many people, you may be able to convince someone at the European Bioinformatics Institute (EBI), National Center for Biotechnology Information (NCBI), Netherlands Bioinformatics Centre (NBIC), or the PSU Galaxy instance to take over. Early decisions about the framework used can have a big impact later on.

## Rule 3: Know Your User Base

The most important component to consider is the Web resource audience. Come up with a use case: when and how will another researcher want to use what you are offering? When you know who you are developing for, many decisions become very straightforward. In our survey, we determined that 36% of Web resource providers think that only researchers with programming experience use their resource. If your audience can manage to run your application on their own computer, let them. It's harder to integrate a Web resource into a scripted workflow.

On the flip side, 64% of resources are also used by researchers without programming experience. They will appreciate a graphical user interface. If you know your users personally, they can give you ideas about how to make the interface fit their needs. Just watching collaborators or students use your software or programs like it will tell you a lot. Get users involved early and include them in the development process. As long as the Web resource is in use, you can solicit feedback from users and see if their needs have evolved (cf. Rule 7).

Constant monitoring of usage patterns and access statistics can be achieved by tracking who visits the Web resource page. If your institution is not already collecting these data from visitors, you can set up a free Web analytics tool within minutes.

Most scientists will come to your Web site via a search engine. Use the indexing power of the search engine spiders by putting, for example, the paper title, abstract, and keywords on the page. When you follow the tips about naming your resource in Rule 1, it should be easy to find.

## Rule 4: Use Services Available to You during Development

The finest way out of much of the strife with hosting and availability is to find someone else to take care of it. If you work on a larger campus or cooperate with someone at an institution that already runs several scientific Web resources, get in touch with the administrators to set up your tool on an established server. Such decisions can greatly influence the software development process. Be aware of the Web address you use to publish your resource. It's best to use a persistent URL or your own domain name for the resource to make sure it is always available under the published address (cf. Rule 1).

Estimate the number of potential simultaneous users. Together with the memory and compute time requirements, this will tell you about the kind of infrastructure you will have to provide to make the resource usable even with many queries coming in at the same time. In an age of high-throughput experiments, this can be a lot. To get an estimate on the number of simultaneous queries your setup can handle, you can perform a stress test, sending a high number of requests with a script from an external source.

If your requirements seem enormous, consider optimizing your program further and finding redundancies between individual queries that can be pre-computed and stored. You can also offer an interface to a cloud-computing on-demand resource, so users are paying for their own computing time. Providing your own large-scale computing infrastructure can be very costly.

You will have to think about a user interface for your resource. Here, an existing framework can save you a lot of time. Examples include Taverna [2], where you provide a description of the input and output in the Web resource description language. Your resource is accessed from a client workbench, in which users can connect your program's output to others to create workflows. It still runs on your own servers and you have to provide the necessary software infrastructure for that.

Galaxy [3] is a customizable workbench that you can download and run on your own Web server. It lets you integrate any command-line tool with a few lines of XML; moreover, it even lets you connect your own tools with the pre-packaged ones to create transparent workflows for your users. You don't need to think about file management and pretty user interfaces, and for those time-intensive jobs, you can easily connect your Galaxy instance to a compute cluster or even run it in the cloud.

If you want to build an interface from scratch, there are also frameworks that make this task easier. Aside from the classic Apache, SQL, and PHP combination, there are a few more modern alternatives: take a look at Ruby on Rails, Tomcat, Pyjamas, or CherryPy.

## Rule 5: Ensure Portability

Make sure that you can still install and run the software on another machine. If you want your software to be available three years from now, consider this strongly. Chances are that the server you are developing on will be replaced or software is updated, which often breaks the functionality. Ensuring portability also makes it easier for computational biologists to install your software locally. Ask a colleague to install the resource from scratch on another computer and you'll see where the pitfalls are.

A brute-force approach to portability is creating a virtual machine (VM). If you have a server where your resource runs just fine, back up its hard disk and restore it in a VM like VirtualBox. That way, you have a running version of your server in a single file. The VM approach is a steamroller tactic for resources with very intricate dependencies. This is a way to provide users with the necessary disk image to run your resource on the compute cloud. However, it is still advisable to provide information on how to set up your program from scratch. Together with source code comments and a high-level user manual, these three layers of documentation will ensure portability.

## Rule 6: Create an Open Source Project

Your source code should be public if the results are used in scientific publications. This is needed for reproducibility (read more about this in Rule 8).

To make your life easier, it is a good idea to place your source code in a repository such as SourceForge.net [4] or Bioinformatics.org [5]. Then you don't have to take care of version control and release issues and it's easier for collaborators to work together over distance. Most of these open source software project sites provide developers a means of communication both with each other and with end users. You can choose between mailing lists (with an online archive), a Web site forum, or an FAQ page.

Many scientists develop programs for one of the proprietary mathematical environments that require expensive licenses to run. If you are still in the planning stage, consider switching to an open source alternative. Your funding body may not be willing to pay for a score of licenses just for the users of your Web resource.

Using open source software, good source code documentation, and standard file formats will go a long way in making your software able to run on other computers (cf. Rules 5 and 7).

## Rule 7: Provide Ample Documentation and Listen to Feedback

A good first impression is very important for Web resources, too. It is crucial that first-time users feel welcome on your site. Provide good documentation and some short info about parameter settings, that is, accepted ranges and optional settings. Ideally, there is a one-click testing possibility with meaningful but easily understood example data. If the output of the example is well-defined, set it up to run periodically as a functional test, for instance during the build process.

Nothing teaches you about parameter settings, file formats, and the general purpose of a resource like a well-crafted demonstration of what it can do, for instance, in a video or screen cast. Many of these points are part of journals' instructions to authors and therefore required when submitting a research article about your Web resource.

A main complaint of the interviewed scientists about working resources was lack of documentation (41%). Beyond the reference to the paper to be cited when using the resource, you should include a brief overview of the resource's purpose, for what kinds of data it is applicable, and pointers to common pitfalls or preprocessing steps that are not so obvious. The latter is hard to imagine beforehand, so find out from users what they consider difficult.

It will be worth your while to set up a channel of communication with your users. Many source code repositories provide such functions (cf. Rule 6), which will save you a lot of time responding to frequent questions users ask about the resource. You can post announcements about maintenance, updates, and bug fixes, and best of all, experienced users often will be there to answer recurring questions raised by newbies, or you can refer them to the collective wisdom of the archives. It is also common practice to provide an e-mail address where the authors can be reached.

Make your life easier by providing a comprehensive error report option that users can click on when something fails, thereby e-mailing you all the information you need to find out what went wrong.

There are two more layers of documentation: in addition to the high-level help for end users, installation instructions will ensure portability, and good source code comments enable you to hand over maintenance responsibility to another developer, maybe even from the user community (cf. Rule 9).

## Rule 8: Facilitate Reproducibility

Reproducibility is always a topic of discussion in computational biology. When a user analyzes data with your Web resource, the results may end up in a research article. Therefore, all the steps needed to reproduce these results have to be documented entirely. In your output, provide users with details about the parameter settings they used, the version number, and information about the input data.

Everything to run the analysis again should be available to reviewers and readers. This includes the source code of the Web resource itself (cf. Rule 6).

It is good practice to make available older versions of the resource for purposes of reproducing results; at least boldly display the Web resource's current version number on the site and hints about how changes may affect the output.

If you change the server's behavior, your users have to know. Even if it is merely a bug fix, be sure to report it publicly in a place that will be noticed when using the server. Keep in mind that some users, for example, may have bookmarked the data submission page.

## Rule 9: Plan Ahead: Long-Term Maintenance

You will probably move to another place during your career. If you leave behind a Web resource, try to make the transition to the new maintainer as smoothly as possible. Ideally, a protocol has already been established during the planning phase (cf. Rule 1). In our survey, we found that more than 24% of Web resources will not be maintained after the original developers leave. Ultimately, it is the responsibility of the senior author of a publication to make sure that this does not happen, but it is a very important consideration for all authors of a Web resource publication.

Documentation of the source code and the installation process will greatly facilitate the transition to new maintainers. If there is no one in your old lab to take over, but the resource is still heavily used, you may be able to convince a current user or a collaborator to take over maintaining the resource. This will be even easier if the program is an open source software project, where a new developer can join at any time.

You may want to take your software with you and find a new home for it. In some circumstances, this requires you to change the Web resource's address. If your resource has been published in a journal, try contacting them and ask to have the link to your resource updated. Some journals may require a formal correction. Get your previous institution to link or forward to the new address from the old page for as long as possible. If you used a persistent URL, all you need to do is update the link (cf. Rule 1).

## Rule 10: Switch off an Unused Resource

During our study, we determined that, while a surprising number of Web resources are still available after a long time, they may not always work any longer. For users, this can be even more frustrating than an unavailable page.

If your resource is no longer under active development, chances are that it has outlived its usefulness after some years. After that, check to see if there is anyone still using it or if the original publication has been cited recently. This should be easy when you followed the advice about collecting statistics in Rule 3. If no one is using your resource any longer, release the source code one last time, and you're done.

If the resource is still useful to some researchers, try posting a notice on the site asking for someone to take over (cf. Rule 9). If all of that seems like too much work and the source code alone won't help anyone, consider creating a VM that runs the resource. When you still have access to the server, this can be done in a matter of hours.

By following these rules, your resource will have a long and productive life.
